# Changes in hippocampal volume and affective functioning after a moderate intensity running intervention

**DOI:** 10.1007/s00429-024-02885-2

**Published:** 2024-12-13

**Authors:** Patrick Klepits, Karl Koschutnig, Thomas Zussner, Andreas Fink

**Affiliations:** 1 University of Graz, Graz, Austria; 2 MRI-Lab Graz, Graz, Austria; 3 BioTechMed-Graz, Graz, Austria

**Keywords:** Hippocampus, Depressive symptoms, VO_2_max, Running

## Abstract

This study examined the effects of a moderately intense seven-week running intervention on the hippocampal volume and depressive symptoms of young men (20–31 years of age) from the general population (*N* = 21). A within-subjects-design involving a two-week baseline period before the running intervention, and two subsequent intervention cycles was applied. At four time points of assessment (t_1_: start of the study; t_2_: end of baseline period/start of the intervention; t_3_: end of the first intervention cycle; t_4_: end of the 2nd intervention cycle/study end) magnetic resonance imaging was performed and symptoms related to depression were assessed employing the Center for Epidemiological Studies Depression (CES-D) Scale. The intervention resulted in a significant increase in the estimated maximum oxygen uptake (VO_2_max), measured with a standardized walking test (average increase from 42.07 ml*kg^− 1^*min^− 1^ to 46.07 ml*kg^− 1^*min^− 1^). The CES-D scores decreased significantly over the course of the running intervention (average decrease from 12.76 to 10.48 on a 20-point scale). Significant volumetric increases in the hippocampus were found, most notably after the first intervention cycle in the left (average increase from 613.41 mm³ to 620.55 mm³) and right hippocampal tail (average increase from 629.77 mm³ to 638.17 mm³). These findings provide new evidence regarding the temporal dynamics of hippocampal changes following engagement in physical activity.

**Supplementary Information:**

The online version contains supplementary material available at 10.1007/s00429-024-02885-2.

## Introduction

In 2002, the global estimated prevalence of physical inactivity was 17% among adults (World Health Organization [WHO] 2002). More than 20 years later, 27.5% of the world’s adult population does not meet the WHO-recommended level of physical activity to ameliorate and maintain their health (WHO 2022). The benefits of being physically active are manifold. For example, 50 min of running per week reduces the total mortality following cardiovascular diseases and cancer by at least 23% (Pedisic et al. 2020). Similarly, regularly engaging in physical activity has been found to reveal positive effects on diverse cognitive and affective functions, especially on stress and depressive symptoms (Gianfredi et al. 2020; Gerber et al. 2013).

Recent meta-analyses summarize the positive effects of physical exercise on affective disorders, including depression (Heissel et al. 2023; Martland et al. 2023). Research has also indicated that exercise interventions reduce symptoms of depression in the general population (Hu et al. 2020). While research with clinical populations is critical, it is also valuable to consider the impact of depressive symptoms in non-clinical samples, as low-level depression symptoms may still have a negative impact on individuals.

There is literature indicating an association between depression and low hippocampal volumes (Brosch et al. 2022; Gray et al. 2020). There are several factors that may help explain the relationship between depression and hippocampal volume, such as stress and the associated glucocorticoid response (McEwen 2006). Glucocorticoid dysregulation is associated with depression, as well as memory problems which are negatively related to hippocampal volume (Travis et al. 2016). However, examining overall volumetric change in the hippocampus may not be sufficient, given that the relationship between hippocampal volume and depression may differ based on hippocampal subfields (Roddy et al. 2019). Concerning the hippocampal subfields, it is unclear which subfields correspond to symptoms related to depression. Some evidence suggests that the hippocampal tail may play a role in depression as well as exercise (Fink et al. 2021; Maller et al., 2018).

There has been additional research regarding aerobic exercise and its relationship to specific hippocampal subfields. In the anterior region of the hippocampus, Thomas et al. (2016) found volumetric increases after six-weeks of cycling followed by a return to baseline after six weeks of rest. A meta analysis by Firth et al. (2018) highlighted positive effects of aerobic exercises (cycling, jogging, walking) on the left hippocampal volume in studies with mainly clinical samples (including mental disorders such as depression) as well as older adults. According to Heissel et al. (2023), aerobic exercises showed especially large effects in reducing depressive symptoms in people who have been diagnosed with depression. Given all these observations, it can be assumed that aerobic exercise is involved with changes in hippocampal subfields and in symptoms related to depression.

There are a limited number of studies investigating the effects of physical activity interventions on brain mechanisms and affective functions, especially in samples of young adults from the general population (Erickson et al. 2019). In one of the rare studies focusing on this demographic, Fink et al. (2021) investigated the effects of a two-week moderately intense running intervention on depressive symptoms and structural changes of the hippocampus. Findings suggest a significant reduction of symptoms related to depression. Importantly, increases in the volume of the hippocampus were observed over the course of the running intervention, and decreases in depressive symptoms were associated with increases in hippocampal volume. The present study was designed to replicate and extend the results of Fink et al. (2021).

Given the aforementioned literature regarding aerobic exercise and the promising results from the Fink et al. (2021) study, the present study also uses moderate running as a form of aerobic exercise. Moderate-intensity exercise is defined by exercising approximately by 30 min a day (Garber et al. 2011). Running is a common exercise intervention in the aforementioned meta-analyses, in addition, it is an accessible form of sport. The present study included a seven-week running intervention with 16 running sessions and four timepoints of measurement. At the beginning and the end of the baseline period, and between both interventional cycles, MRI scans were performed, and depressive symptoms were measured via the CES-D (Hautzinger et al. 2012). In addition, at the beginning and the end of the intervention, two walking tests were performed to estimate the VO_2_max. While during the baseline period, no substantial changes in depressive symptoms and hippocampal volume should be evident, the central hypothesis of this study is that engagement in the running intervention reduces symptoms related to depression and increases the volume of the hippocampus. Additionally, it is hypothesized that increased fitness-level will be negatively associated with depressive symptoms. The current study increased the frequency of measurements and had a longer duration than the Fink et al. (2021) paper, as well as including a rough estimate of VO_2_max (maximum oxygen uptake). By applying several time-points of measurement this study also aims to investigate whether the length of the exercise intervention impacts hippocampal and corresponding changes in affective functions, as discussed by Fink et al. (2021). Therefore, additional assessments between both intervention cycles enable the exploration of possible linear, cubic, and polynomial trends of changes in hippocampal volume and symptoms related to depression.

## Methods

The present study utilized a comprehensive within-subjects approach involving three critical study periods and four time points of brain imaging and psychometric assessment. The comparison of a baseline period (no physical activity) with two following intervention cycles (supervised running interventions) should reveal important new insights into the time course of changes in affect and brain structure following engagement in a moderately intense running intervention.

Second, measuring objective fitness parameters allows additional insights into the physical benefits of the running intervention. Therefore, an estimation of the maximum oxygen uptake (VO_2_max) was included in the study before and after the running intervention. VO_2_max was estimated via a walking test developed by the Urho Kekkonen Fitness Institute Foundation (UKK Walking-Test; Oja et al. 2013).

Third, it is not clear from the literature which specific regions of the hippocampus are most sensitive to physical activity. This study utilizes MRI scans in order to examine the effects of running on different subfields of the hippocampus, as assessed by the FreeSurfer processing pipeline.

### Participants

Recruitment for the study began in January of 2022, and the study began in April. The study was advertised online via social media (Facebook, Instagram, YouTube), a web recruitment platform, and through university email. Physical advertisements including posters, cards, and flyers were displayed in the host university and in public areas. Information regarding the study was also presented in lectures at various universities in the city. Financial compensation was offered to participants. Psychology students at the host university had the option to receive course credit as an alternative form of compensation. Participants were also offered the opportunity to receive the de-faced, anonymized MRI scan of their brain.

**Fig. 1:**
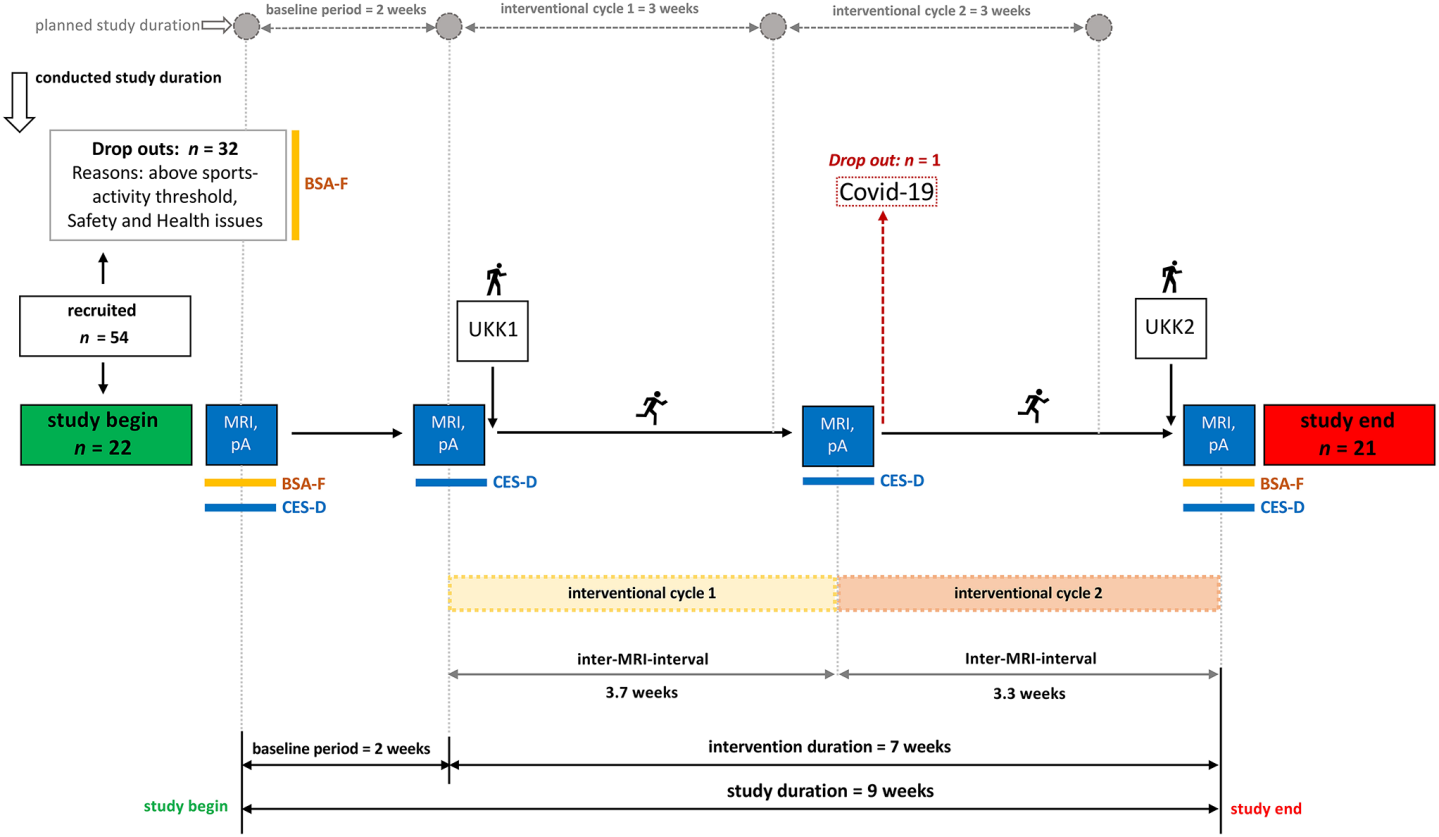
Study design. Note Flow diagram and study design. The study involved four different time points of measurement including magnetic resonance imaging and psychometric assessments. For estimation of the VO_2_max a UKK-Walkingtest (Oja et al. 2013) was performed at the beginning and the end of the running intervention, respectively. In the head of the figure, the planned study duration is outlined, which was slightly different from the actual duration of the interventions

Fifty-four men from the general population (mostly university students) without psychiatric or neurological disorders registered for the study. In order to meet the requirements for an additional study which used the same participant data, a male-only sample was used. Figure 1 summarizes the study design and the recruitment process of this study. Firstly, participants were screened for general study-eligibility (like MRI-safety). Handedness was not part of the eligibility criteria, since previous research indicates that lateral differences between the left and right hippocampus should be independent of handedness (Guadalupe et al. 2017; Kong et al. 2022.) Therefore, the sample was mixed in handedness, 17 out of 21 total participants were right-handed (as determined by pre-screening). An important inclusion criterion was that participants engaged in less than 150 min of regular physical exercise or sports activities per week, which corresponds with the recommended WHO levels of moderate physical activity (WHO 2020). Potential participants were screened for activity levels using a sports activity questionnaire (Bewegungs- und Sportaktivität Fragebogen; BSA-F; Fuchs et al. 2015). According to all these requirements, 22 people were selected from the 54 study-applicants. Written informed consent was obtained. The final sample consisted of 21 males between 20 and 31 years (*M* = 24.50, *SD* = 3.49). The study was approved by the authorized ethics committee (GZ. 39/24/63 ex 2021/22). Participants who completed the study received either financial compensation (*n* = 20) or course credits (*n* = 1). The number of participants was nearly in line with the planned sample size of 23 to detect small to moderate time effects (Cohen’s *f* = 0.25; α = 0.05; a priori power = 0.80; number of groups = 1; 4 measurements; correlation among repeated measures = 0.5; G*Power 3.1.9.7; Faul et al. 2007), based on the results of previous literature (Erickson et al. 2011; Fink et al. 2021).

### Study design and procedure

**Fig. 2:**
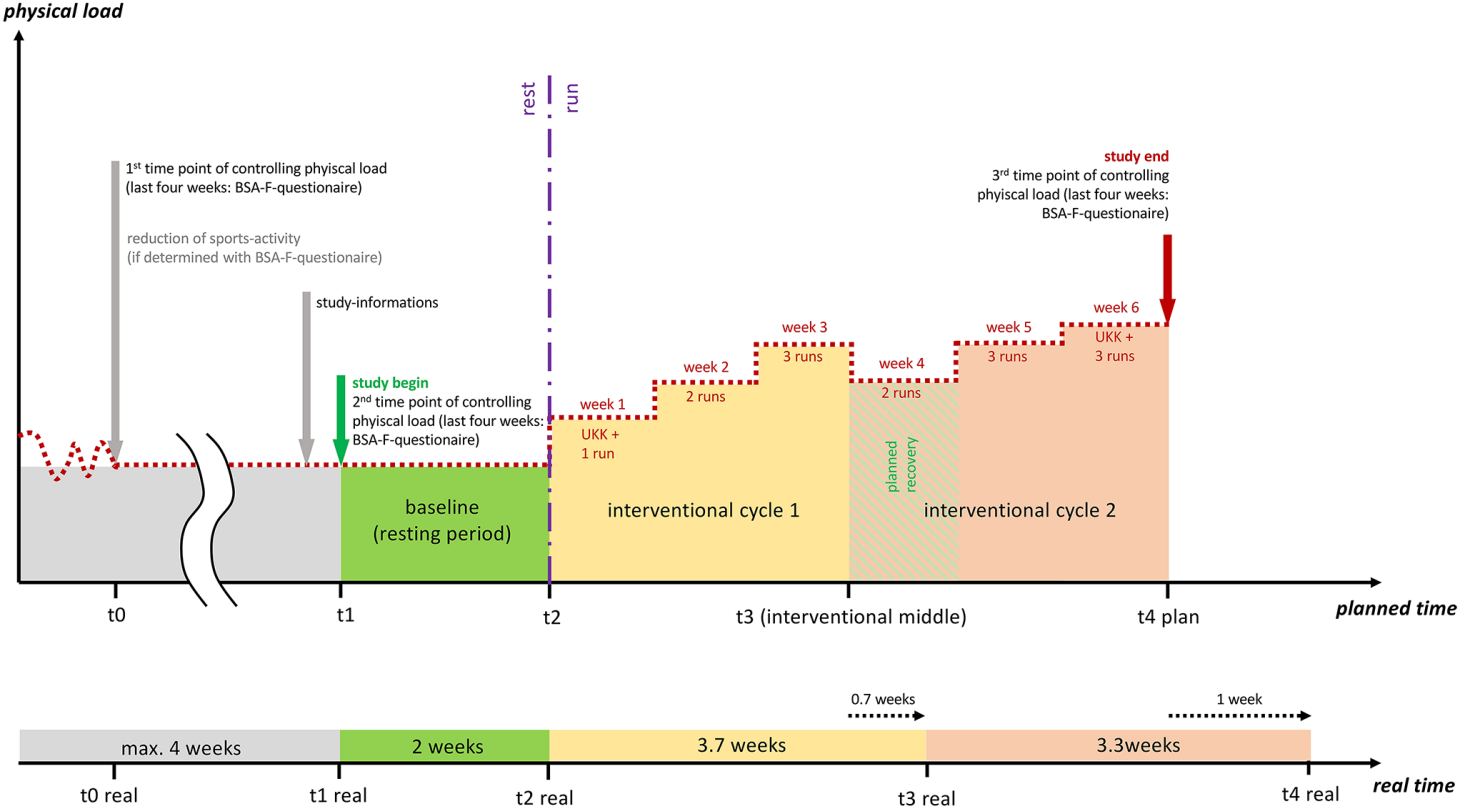
Intervention plan. Note Grey colors indicate the pre-study phase which controlled the physical activity level. The green areas represent the baseline phase of the study. Yellow and red areas show the interventional cycles. The dotted red line frames the estimated physical activity level (physical load as y-Axis). The vertical violet line indicates the temporal border between the resting and running phases. The hatched green area in interventional cycle 2 shows a training-deload phase

This study involves a comprehensive within-subjects design with a two-week baseline period, and two running intervention cycles, which were planned for three weeks each (Fig. 2). Over the study period, there were four-time points of assessment (MRI, psychometric assessment) and two UKK-Walkingtests (Oja et al. 2013) for estimation of the VO_2_max at the beginning and the end of the intervention. The duration of the baseline period was planned for two weeks based on the waiting control group period described by Fink et al. (2021). The duration of the intervention was planned for six weeks since previous literature which utilized an exercise intervention of this length demonstrated volumetric increases in the anterior hippocampus over the intervention period (Thomas et al. 2016). This longer duration of six weeks, in comparison to the two weeks intervention period of Fink et al. (2021), enabled an additional timepoint of measurement. Due to challenges related to the pandemic situation, the duration of the intervention was extended to seven weeks (Fig. 1).

#### Intervention

Before starting the running intervention, the individual pace was guided by estimating the maximum oxygen volume (VO_2_max) with the UKK-Walkingtest (Oja et al. 2013) and a following 400 m test run on a standardized cinder track. According to the literature, the heart rate at the end of the UKK-Walkingtest (HRUKK) corresponds to approximately 80% of the maximum individual heart rate (Laukkanen et al. 2000.) All participants underwent real-time heart rate monitoring by the experimenter in the test run, intending to keep the heart rate beneath HRUKK. Hence, the elapsing time for the test run following the HRUKK–threshold guided the participant to determine their pace for the upcoming running interventions. Given the long duration of the running intervention, it was important to maintain study commitment and motivation. Therefore, participants were free to choose their own running routes, provided that their route had the same major characteristics as the standardized running route.

The standardized running route was located in a mostly forested local recreation area and consisted of a 5-kilometer distance and an elevation change of about 100 m. Approximately one running session per week had to be done in the group setting on this standardized route. The group sessions were offered nearly every day with up to three experimenters. During the sessions, experimenters used a pulse oximeter to monitor the pace of individuals. The purpose of the pulse monitoring was solely to ensure that individuals maintained a moderate running pace (which was unique for each participant) and as such, these measurements were not recorded. Depending on the group size and individual running speed (pace), the number of experimenters varied. The distances between the participants and the experimenter were kept low in order to allow for proper running supervision (regular monitoring of heart rate, pace, and running technique). Each participant engaged in approximately 16 running sessions, excluding the two UKK-Walkingtests (Oja et al. 2013). Figure 2 summarizes the planned timetable of the running intervention, along with the actual durations.

For each participant, it was possible to adapt the running dates individually in compliance with the required regeneration phase of about 48 h (Hofmann and Tschakert 2017). When possible, sessions were conducted between noon and evening for standardization purposes.

For each running session, participants recorded individual pace, time, distance and elevation change (to ensure adherence to the standardized track characteristics). The running sessions were recorded through a smartphone app as well as in a booklet. Each run was self-paced and lasted approximately between 30 and 40 min with an additional 20 min for warm-up, cool-down, and smartphone-assessment (data for another study). All instructions were held on the very first group running session and were available on YouTube.

### Psychometric tests

One focus of this study was the psychometric evaluation of intervention-related changes of symptoms related to depression, which were assessed by means of the German version of the Center for Epidemiological Studies Depression Scale (CES-D; Hautzinger et al. 2012). According to Radloff (1977) the CES-D is designed to assess symptoms related to depression in the general population, and was suitable for assessing symptoms related to depression the young male sample of the general population in this study. This brief self-report scale covers depressive symptoms in the emotional, motivational, cognitive, somatic, and motoric domain, and serves as “a first-stage screener to target respondents with depressive symptoms for more in-depth clinical assessment” (Vilagut et al. 2016, p.13). The CES-D contains 20 items. For each question, participants are asked about the frequency in which they experienced a certain symptom during the past week, and select a response from a 4 point Likert scale, ranging from “infrequent” (0) to “most of the time” (3). According to the test CES-D test-manual, a cut-off score of 22 is suggested for further diagnostic clarification (Hautzinger et al. 2012). The CES-D was the first questionnaire in the psychometric testing battery and lasted about five minutes. The mean CES-D score in the first timepoint of assessment was 13.48 (*SD* = 7.88; Skewness = 0.65, *SE* of skewness = 0.50), which is very similar to the data of representative samples from the general population (Hautzinger et al. 2012). Legal copyright restrictions prevent public archiving of these questionnaires, which can be obtained from the copyright holders in the cited references.

### MRI assessment and analysis

#### Intervention

MRI data was acquired on a 3.0 T MAGNETOM Vida system with Syngo MR XA31 (Siemens Healthineers, Erlangen, Germany) using a 64-channel head coil. T1-weighted measurements were obtained using a 3D MPRAGE-sequence with the following parameters: 176 sagittal slabs [1.00 mm slice thickness], FoV read 256 mm, TR 2530 msec, TE 3.88 msec, base resolution 256 [acquisition matrix 256 × 256, 1.00 mm iso-voxel, no partial fourier], flip angle 7 deg, phase encoding direction AP, phase oversampling 0, PAT 2 [GRAPPA, accel. factor PE 2], single shot, interleaved, bandwidth 190 Hz/Px. The measurement time for this sequence was 6 min 03 s.

#### MRI data processing

After the removal of facial features (https://github.com/poldracklab/pydeface/tree/master/pydeface), all T1-weighted images were reviewed with the MRI Quality Control tool (MRIQC; Esteban et al. 2017). MRIQC reports of each image are included in the derivatives/mriqc folder on openneuro (in order to maintain an anonymized review process, we can only send the openneuro link upon request, as the authors are mentioned by name).

The structural T1-weighted 3D datasets were processed using the longitudinal Freesurfer pipeline (https://surfer.nmr.mgh.harvard.edu/ version 7.3.2 8.8.2022). FreeSurfer is a freely available software for the processing of neuroimaging data, including automatized, standardized, and well-proven algorithms and routines for image processing for both cross-sectional and longitudinal research designs (full documentation of this software can be found at https://surfer.nmr.mgh.harvard.edu/). FreeSurfer also includes processing tools for the automated segmentation of subfields of the hippocampus (Iglesias et al. 2015), which is of central interest in this study. Longitudinal image data were processed cross-sectionally for all four-time points (t_1_, t_2_, t_3_, t_4_) following the default FreeSurfer workflow, including multiple time points per subject (“recon-all”). In the first step, all sets of two-time points (e.g., participant-1, t_1_ t_3_) were calculated independently with the longitudinal stream in FreeSurfer (“recon-all -long”) followed by the computation of individual intra-subject templates (Reuter et al. 2012). Afterwards, the results of the longitudinal calculation were compared in native space. In a second step, we performed the longitudinal hippocampal subfield segmentation (“segment”) based on the work of Iglesias et al. (2015) and Iglesias et al. (2016). The Freesurfer tool is based on an atlas of statistical probability generated with ultra-high resolution ex vivo MRI data for automated hippocampal subfield allocation on the subject level. The method also segments the nuclei of the amygdala, further increasing segmentation accuracy, since overlapping between structures is minimized (Saygin et al. 2017). The hippocampal subfields can be summarized into the main hippocampal parts of head (parasubiculum, presubiculum-head, subiculum-head, CA1 head, CA3 head, CA4 head, GC ML DG head, molecular layer head, HATA), body (presubiculum body, subiculum body, CA1 body, CA3 body, CA4 body, GC ML DG body, molecular layer body, fimbria), and tail.

### Statistical analysis

To investigate the effects of the running intervention on symptoms related to depression (CES-D scores), General Linear Models (GLM) for repeated measures with the TIME point of assessment as a within-subjects factor (t_1_, t_2_, t_3_, and t_4_) were conducted for the head, body and the tail of the hippocampus. In addition, GLMs were computed for each subfield of the head and the body. The factor TIME involves the baseline period and both intervention cycles, respectively: t_1_ to t_2_: baseline; t_2_ to t_3_: interventional cycle 1; t_3_ to t_4_: interventional cycle 2). In case of significant GLM effects, follow-up paired t-tests were computed to test significant differences between the TIME points of assessment. In addition, polynomial contrasts were tested. These additional contrast analyses further allowed us to assess whether the time-related changes in the hippocampal volume follow linear, quadratic, or cubic functions.

In case of violations of sphericity assumptions, degrees of freedom were Greenhouse-Geisser corrected. The significance level was *p* <.05 in all statistical analyses. For the one-way GLM repeated measures analyses, estimates of effect sizes are given in terms of eta-squared measures (η^2^). For the *t*-tests, Cohen’s *d* values are reported. A Pearson correlation was conducted to assess the association between the fitness-improvement due to running (operationalized via the estimates of VO_2_max) and the reduction of depressive symptoms. All statistical analyses were performed with the statistical software SPSS version 28.0.0.0 (IBM Corp. Released 2021).

## Results

Table 1 summarizes the characteristics of the final sample (*N* = 21) at baseline.

**Table 1:** Summary characteristics of the sample at baseline

Characteristics	t_1_ (Baseline)
Gender, male	21 (100%)
Age, years	24.50 (3.49)
Caucasian	20 (95.24%)
Current smoker	2 (9.52%)
Students	19 (90.48%)
Handedness	
**Right-handed**	17 (80.95%)
**Left-handed**	3 (14.29%)
**Ambidextrous**	1 (4.76%)
BSA-F, min/week	
**Running**	0 (0.00)
**Other sports related activities**	110.34 (161.83)
BMI, kg/m²	24.61 (3.18)

Note. Results are expressed as number (%) or mean (standard deviation). This baseline data refers to the first time-point of measurement for the final sample (*N* = 21). BSA-F represents the sports activity level of the sample. BMI represents the mean body mass index of the sample. BSA-F and BMI were re-measured at the final timepoint. From t_1_ to t_4_, mean BSA-F values changed from 0 (*SD* = 0) minutes running per week and to 76 (*SD* = 15) minutes running per week, and mean values of other sports activity changed from 110.34 (*SD* = 161.83) to 94.40 (*SD* = 111.48) minutes of other sports related activity per week. The mean BMI value changed from 24.61 (*SD* = 3.18) to 24.52 (*SD* = 3.10)

### Intervention effects on symptoms related to depression (CES-D scores)

**Fig. 3:**
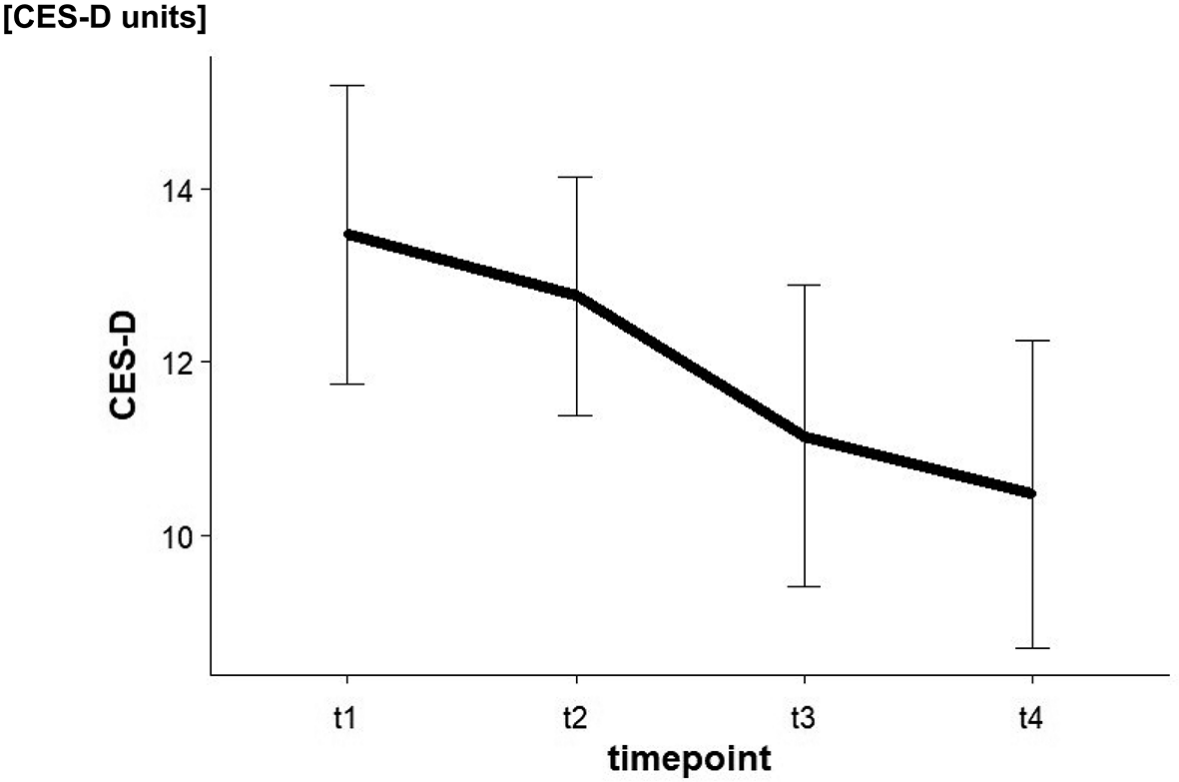
CES-D scores. Note CES-D Scores at t_1_, t_2_, t_3_, and t_4_. The lowest CES-D value corresponds to the value of zero (0), the highest value is 60 (see 2.3). The units of the CES-D score are defined with the value one (1). The more depressive symptoms occur, the higher the value. Error bars indicate ± 1 SE

The repeated measurement GLM for the CES-D involving the four-time points of assessment revealed a significant TIME effect (*F*(3,60) = 3.55, *p* =.019, η^2^ = 0.15). Paired *t*-tests indicated significant reductions of CES-D scores between t_1_ and t_4_ (*t*(20) = 2.58, *p* =.018, *d* = 0.56), and between t_2_ and t_4_ (*t*(20) = 2.20, *p* =.040, *d* = 0.48), respectively. The contrast analysis revealed a significant linear trend of the reduction of symptoms related to depression (*F*(1,20) = 6.98, *p* =.016, η^2^ = 0.26). The time-course of the CES-D scores is summarized in Fig. 3.

### Intervention effects on the volume of the head, body, and tail of the hippocampus

Significant TIME effects were found for the left (*F*(3,60) = 3.475, *p* =.021, η^2^ = 0.15) and right hippocampal tail (*F*(3,60) = 3.539, *p* =.020, η^2^ = 0.15), respectively. Follow-up paired t-tests showed significant increases after the first intervention cycle for the left (*t*(20) = -2.32, *p* =.031, *d* = − 0.51) and right hippocampal tail (*t*(20) =-2.23, *p* =.037, *d* =-0.49). Over the course of the intervention, significant increases were shown in the left hippocampal tail (*t*(20) =-2.89, *p* =.009, *d* =-0.63). Significant decreases were found for the left hippocampal tail in the baseline-period (t_1_ to t_2_: *t*(20) = 2.63, *p* =.016, *d* = 0.57) and for the right hippocampal tail in the second intervention cycle (t_3_ and t_4_: *t*(20) = 2.42, *p* =.025, *d* = 0.53). Contrast analysis revealed a significant quadratic trend for the right hippocampal tail (*F*(1,20) = 5.25, *p* =.033, η^2^ = 0.21). Significant cubic trends were found for the left (*F*(1,20) = 5.271, *p* =.033, η^2^ = 0.21) and right hippocampal tail (*F*(1,20) = 4.36, *p* =.0499, η^2^ = 0.179).

**Fig. 4:**
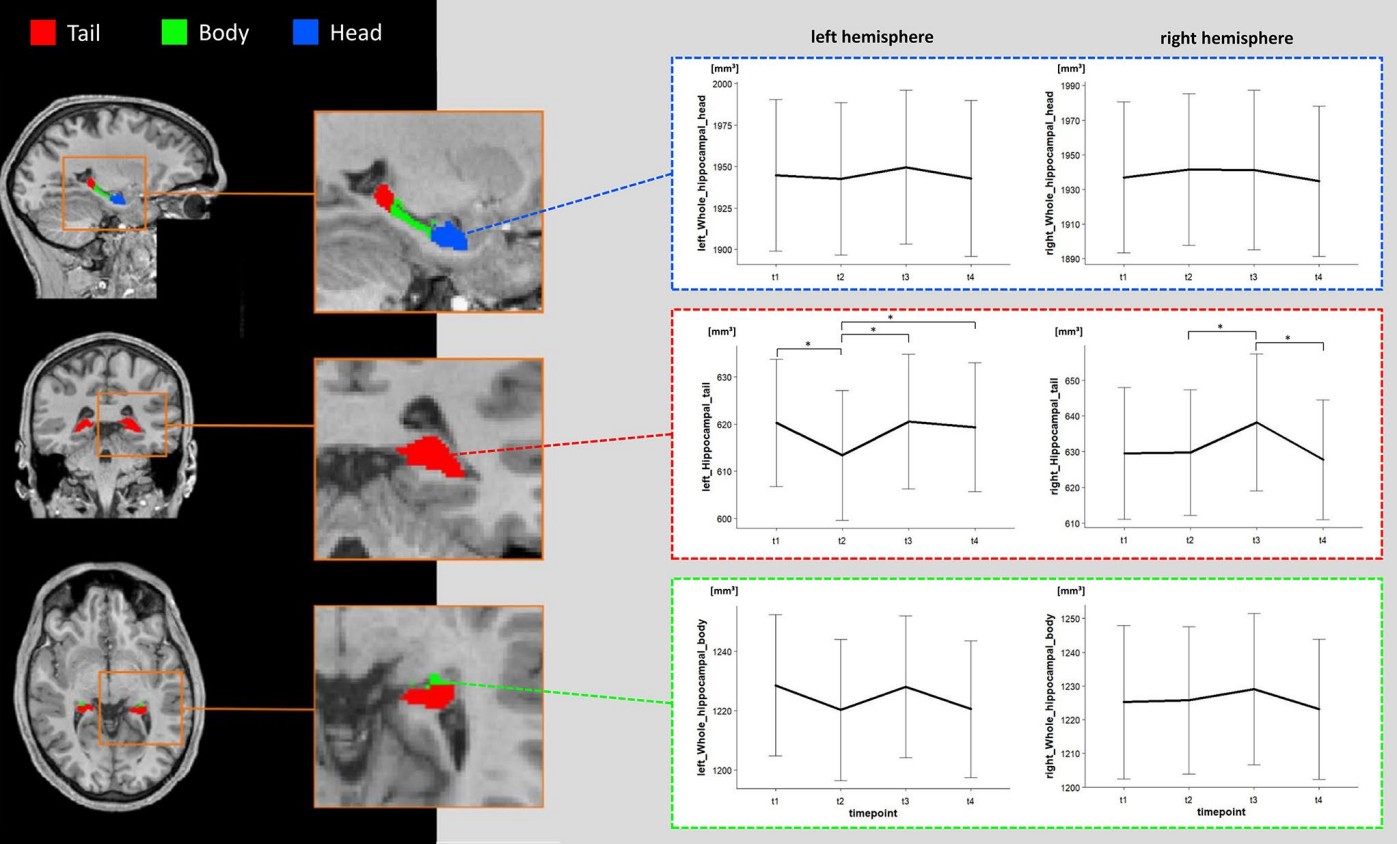
Volumetric Changes in the hippocampal head, body, and tail. Note Changes in mm³ of the volume of the head (blue), body (green), and tail (red) of the hippocampus over the study period. Significant TIME-related changes were only significant in the left and right hippocampal tail. Significance of *p* <.05 is indicated by *. Error bars indicate ± 1 SE

No significant TIME effects were found for the hippocampal body (left: *F*(3,60) = 2.034, *p* =.119, η^2^ = 0.09; right: *F*(3,60) = 0.423, *p* =.737, η^2^ = 0.02) and head (left: *F*(3,60) = 0.437, *p* =.727, η^2^ = 0.02; right: *F*(3,60) = 0.336, *p* =.799, η^2^ = 0.02). The volumetric changes of hippocampal head, tail and body are summarized in Fig. 4.

### Volumetric changes in the subfields of the head and the body of the hippocampus

Separate GLM analyses of the subfields of the left and right hippocampal head revealed one significant TIME effect in the left HATA (*F*(2.28,45.52) = 3.63, *p* =.029, η^2^ = 0.15). Follow-up paired *t*-tests showed significant volumetric decreases of the left HATA during the intervention (t_2_ to t_4_: *t*(20) = 5.12, *p* <.001, *d* = 1.12; t_1_ to t_4_: *t*(20) = 2.12, *p* =.047, *d* = 0.46; t_2_ to t_3_: *t*(20) = 2.24, *p* =.037, *d* = 0.49). Contrast analysis revealed a significant linear trend for the left HATA (*F*(1,20) = 8.58, *p* =.008, η^2^ = 0.30). In addition, the TIME effect was significant for the left GC ML DG body (*F*(3,60) = 3.135, *p* =.032, η^2^ = 0.14). Subsequent paired t-tests showed significant volumetric increases of the left GC ML DG body during the first interventional cycle (t_2_ to t_3_: *t*(20) = -3.31, *p* =.003, *d* = − 0.72). A significant cubic trend was determined by contrast analysis for the left GC ML DG body (*F*(1,20) = 9.35, *p* =.006, η^2^ = 0.319). A table detailing volumetric changes in the three major hippocampal parts (tail, body, head) with the significant corresponding subfields (HATA, GC ML DG body) are summarized in supplementary Table 1.

### Associations of changes of CES-D scores with changes in VO2max

**Fig. 5:**
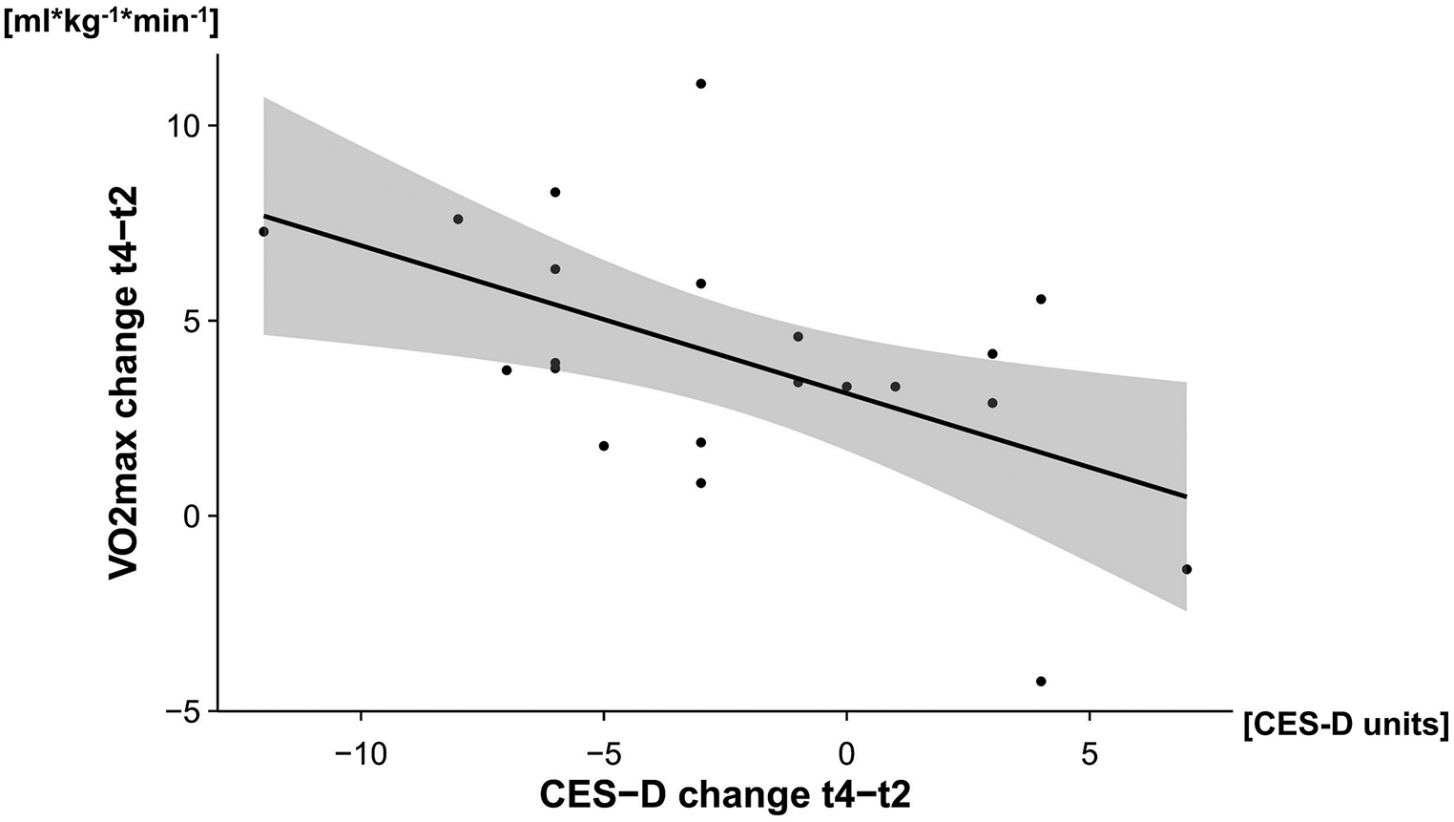
Correlation of VO _2_ max with CES-D. Note Pearson-correlation of the score-differences between VO_2_max and CES-D. Both scores represent the differences between t_4_ and t_2_. The grey area indicates a confidence interval of 95%

A significant correlation between changes in the estimated maximum oxygen uptake (VO_2_max) and the changes in CES-D scores over the course of the intervention (t_2_ to t_4_) was observed (*r*(19) = − 0.541, *p* =.011). Accordingly, an increase in VO_2_max was significantly associated with reductions in the CES-D scores (Fig. 5). Table 2 summarizes the estimated marginal means and the standard errors for CES-D and VO_2_max at each timepoint of the study.

**Table 2:** CES-D and VO _2_ max values

	CES-D scores		VO_2_max	
Timepoint	*M*	*SE*	*M*	*SE*
t_1_	13.48	1.72		
t_2_	12.76	1.37	42.07	1.74
t_3_	11.14	1.74		
t_4_	10.48	1.77	46.07	1.53

Note. *M* = Estimated Marginal Mean, *SE* = Standard Error of Mean. CES-D scores were measured using a 20-item scale, with higher values indicating more symptoms related to depression. VO_2_max scores represent the maximum oxygen volume in ml*kg^− 1^*min^− 1^, estimated with the UKK-Walking-test (t_2_ and t_4_)

## Discussion

Regular running over seven weeks resulted in significant reductions of symptoms related to depression, significant increases of the estimated maximum oxygen reuptake (VO_2_max), and significant volumetric changes in the left and right hippocampal tail. Specifically, volumetric increases in the left (1.16%) and the right hippocampal tail (1.34%) were observed during the first intervention cycle. After the second intervention cycle, the right hippocampal tail showed significant volume reductions (1.66%), whereas the left hippocampal tail exhibited no significant changes during that time. The significant intervention-related increases in the hippocampal tail and the decreases in symptoms related to depression replicate the results of Fink et al. (2021) and align with previous evidence in this field that certain regions of the hippocampus can change in response to exercise (Erickson et al. 2011; Firth et al. 2018; Frodl et al. 2020; Thomas et al. 2016). Interestingly, after the first intervention cycle, the volume of the left GC ML DG body increased significantly (2.59%). Although there was an overall pattern of volumetric increase, the left HATA (a subfield of the hippocampal head) decreased its volume over the course of the running intervention by 2.76%.

Throughout the course of this study, significant changes in affective, neuronal, and fitness-related measures were revealed. The most significant differences were observed after the first intervention cycle. After the second intervention cycle, no further significant changes in symptoms related to depression were observed. Due to the comparatively low CES-D scores in the general population and in the present sample (t_4_: *M* = 10.48; *SD* = 8.12), potential floor effects can be assumed. Another possible reason that symptoms related to depression decreased and then plateaued could be dose-specific effects. A systematic review by Pearce et al. (2022) observed that initial engagement in physical exercise was associated with a substantial decrease in depression risk. However, as the amount of physical activity increased, additional risk reduction effects on depression were minimal. Looking at the second intervention cycle of this study, higher cumulated levels of physical activity over several weeks may show similar diminishing effects on the reduction of symptoms related to depression.

On the neuronal side, both the left and the right hippocampal tail showed significant volumetric increases after the first intervention cycle, which remained stable after the second interventional cycle for the left tail, and decreased in the right tail, respectively. These results are in line with studies which likewise identified the hippocampal tail as being critically implicated in the development and remission of depression (Maller et al. 2018; Nogovitsyn et al. 2020). Interestingly, the volumes of the left hippocampal tail and the left GC ML DG body followed a similar curvilinear pattern of changes (significant cubic contrasts) during the intervention. Taken together, these findings clearly indicate that intervention-related changes in the hippocampus may follow a complex non-linear time course, a finding that must be addressed more rigorously in future research. The findings also suggest that it is necessary to look at specific subfields of the hippocampus in investigating the effects of physical activity.

The finding that specifically the left GC ML DG (DG = dentate gyrus) body showed intervention-related increases appears to be noteworthy in the context of the ongoing discussion on adult hippocampal neurogenesis (Liu 2022). According to exercise experiments in animals, neurogenesis can occur specifically in the dentate gyrus (e.g., DiFeo and Shors 2017; Gao et al. 2023). A comprehensive overview of the development stages of new neurons during hippocampal neurogenesis in animals, which occurs, for example, in the granule cell layer and molecular layer, is illustrated in the review of Gao et al. (2023). Specifically, animal models suggest that particularly the insulin-like growth factor-1 (IGF-1) mediates the effects of brain-derived neurotrophic factors (BDNF) and promotes exercise-induced neurogenesis (Voss et al. 2011). These BDNF-levels should also play an important role in both physical activity (Voss et al. 2011) and depression (Sheline 2011). Unfortunately, to our knowledge no studies involving participants from the general population have investigated these phenomena. Therefore, additional measurement of BDNF-levels is recommended for future research. In addition to BDNF-levels, reduced glucocorticoid level after exercise may also decrease symptoms related to depression (Nabkasorn et al. 2006). This could also be a possible mechanism underlying the relationship between exercise, hippocampal volume and depressive symptoms. Somewhat contrary to our expectations, significant volumetric decreases (1.11%) were found in the left hippocampal tail after the baseline period (period before the running intervention). One possible explanation for this finding could be the social isolation during the lockdowns and related social restrictions in the time before the study started. A recent work by Lammer et al. (2023) investigated the effects of social isolation on more than 1,400 healthy people, participating in a baseline and an approximately six-year follow-up measurement. The findings of this study indicated that social isolation leads to grey matter loss in the hippocampus (Lammer et al. 2023). In addition, there were also some effects of social isolation on the functional characteristics of the brain, especially in the hippocampus (Zajner et al. 2022).

The decrease of the left HATA volume (2.76%) throughout the intervention seems to be contrary to the literature, which suggests increases in the hippocampal volume after regular physical activity (e.g. Erickson et al. 2015; Fink et al. 2021; Firth et al. 2018; Wilckens et al. 2021). Decrease of HATA volume also seems unusual given that smaller hippocampal volume is associated with depression, whereas CES-D scores decreased throughout the present study.

However, the HATA subfield in particular may have an atypical relationship with depressive symptoms compared to the rest of the hippocampal subfields. A meta-analysis by Sun et al. (2023) included several studies which measured the HATA. The authors stated that people with major depressive disorder had larger volumes in the right HATA, whereas all other subfields of the hippocampus were smaller (with the exception of the parasubiculum). No significant relationship was observed between the left HATA and depression.

The relationship between the HATA and depression may be further explained by the connection of the HATA to the amygdala. Fastenrath et al. (2014) observed that during the encoding of negative emotionally arousing information, the connection between the hippocampus and the amygdala increased. Hence, future research could focus on volumetric changes in HATA in the broader context of its connectivity with the amygdala. It is possible that the decrease in HATA volume could play a role in reducing negative emotional arousal, which may be partially associated with the decrease in the CES-D scores.

### Limitations

The pandemic situation during the study period influenced the running intervention in several important ways. Only a comparatively low number of *N* = 21 participants could be recruited, which certainly limits the power of this study. In addition, the second intervention cycle was impacted in terms of study organization (delayed assessments due to COVID-19 in the study personnel). Therefore, the results in the second intervention cycle should be interpreted with some caution due to the unexpected temporal fluctuations. Participants were uncertain of when the study would end, which could have potentially caused distress. In light of this circumstance, a recent review showed an association between uncertainty and mental health problems (Massazza et al. 2023). To summarize, this work not only highlights the positive effects of running during the COVID-19 pandemic, but also the potential negative impacts of uncertainty, which were reflected more broadly in society during that time (Barchielli et al. 2022). The inclusion of a control group without exercise would have been ideal to control for any time related changes in the outcome measures. Unfortunately this was not possible for economic reasons. The present study also included an estimate of VO_2_max via a walking test, as opposed to a direct measurement using a spiroergometry. The UKK walking test (Oja et al. 2013) is an inexpensive, validated measure (Zakarias et al. 2003) that is accessible for people who do not exercise frequently. Measuring VO_2_max directly is cost-intensive, and it was not deemed essential given that this is an exploratory study focusing on MRI measurements. Future research can be conducted using spiroergometry in order to get a more precise understanding of how VO_2_max changes over the course of exercise interventions, and how this relates to depressive symptoms and changes in hippocampal volume.

It is also important to note that our study involved running in natural forested settings. Literature regarding green exercise demonstrates that this may contribute to the reduction of depressive symptoms (Li et al. 2022). Future research regarding exercise and depression should hence be mindful of the setting in which the exercise intervention takes place.

In terms of the quality of the MRI-images, T2-weighted images with higher resolution, in addition to the used T1-weighted images, could increase the precision of the subfield segmentations. Besides these optimizations, additional sequences determining diffusivity, connectivity, and metabolic and functional changes due to the running intervention could help integrate these findings into the discussed biological mechanisms such as the dentate gyrus (e.g., GC ML DG body) and HATA.

### Conclusion

To conclude, this study demonstrates specific benefits of regular physical activity on symptoms related to depression and hippocampal volume. The seven week long running intervention was associated with a reduction in symptoms related to depression. Regarding the hippocampus, the volumetric changes seem to be very specific and localized to different subfields. Hence, to understand these volumetric changes in a clearer way, future investigations into underlying functional (connectivity analysis), metabolic (e.g. BDNF) and neuroendocrinal mechanisms (Roozendaal & Gaugh, 2011) are needed.

## Electronic supplementary material

Below is the link to the electronic supplementary material.

Supplementary Material 1

## Data Availability

Data can be requested from the corresponding authors.

## References

[CR1] Barchielli B, Cricenti C, Gallè F, Sabella EA, Liguori F, Da Molin G, Liguori G, Orsi GB, Giannini AM, Ferracuti S, Napoli C (2022) Climate Changes, Natural resources Depletion, COVID-19 pandemic, and russian-ukrainian war: what is the impact on habits Change and Mental Health? Int J Environ Res Public Health 19(19):3–18. 10.3390/ijerph191911929 10.3390/ijerph191911929 PMC9565033 36231229

[CR2] Brosch K, Stein F, Schmitt S, Pfarr JK, Ringwald KG, Thomas-Odenthal F, Meller T, Steinsträter O, Waltemate L, Lemke H, Meinert S, Winter A, Breuer F, Thiel K, Grotegerd D, Hahn T, Jansen A, Dannlowski U, Krug A, Kircher T (2022) Reduced hippocampal gray matter volume is a common feature of patients with major depression, bipolar disorder, and schizophrenia spectrum disorders. Mol Psychiatry 27(10):4234–4243. 10.1038/s41380-022-01687-4 35840798 10.1038/s41380-022-01687-4 PMC9718668

[CR3] DiFeo G, Shors TJ (2017) Mental and physical skill training increases neurogenesis via cell survival in the adolescent hippocampus. Brain Res 1654:95–101. 10.1016/j.brainres.2016.08.015 27531182 10.1016/j.brainres.2016.08.015

[CR4] Erickson KI, Voss MW, Prakash RS, Basak C, Szabo A, Chaddock L, Kim JS, Heo S, Alves H, White SM, Wojcicki TR, Mailey E, Vieira VJ, Martin SA, Pence BD, Woods JA, McAuley E, Kramer AF (2011) Exercise training increases size of hippocampus and improves memory. Proc Natl Acad Sci USA 108(7):3017–3022. 10.1073/pnas.1015950108 21282661 10.1073/pnas.1015950108 PMC3041121

[CR5] Erickson KI, Hillman CH, Kramer AF (2015) Physical activity, brain, and cognition. Curr Opin Behav Sci 4:27–32. 10.1016/j.cobeha.2015.01.005

[CR6] Erickson KI, Hillman C, Stillman CM, Ballard RM, Bloodgood B, Conroy DE, Macko R, Marquez DX, Petruzzello SJ, Powell KE (2019) Physical activity, cognition, and brain outcomes: a review of the 2018 physical activity guidelines. Med Sci Sports Exerc 51(6):1242–1251. 10.1249/MSS.0000000000001936 31095081 10.1249/MSS.0000000000001936 PMC6527141

[CR7] Esteban O, Birman D, Schaer M, Koyejo OO, Poldrack RA, Gorgolewski KJ (2017) MRIQC: advancing the automatic prediction of image quality in MRI from unseen sites. PLoS ONE 12(9):e0184661. 10.1371/journal.pone.0184661 28945803 10.1371/journal.pone.0184661 PMC5612458

[CR8] Fastenrath M, Coynel D, Spalek K, Milnik A, Gschwind L, Roozendaal B, Papassotiropoulos A, de Quervain DJF (2014) Dynamic modulation of amygdala-hippocampal connectivity by emotional arousal. J Neurosci 34(42):13935–13947. 10.1523/JNEUROSCI.0786-14.2014 25319690 10.1523/JNEUROSCI.0786-14.2014 PMC6705297

[CR9] Faul F, Erdfelder E, Lang AG, Buchner A (2007) G*Power 3: a flexible statistical power analysis program for the social, behavioral, and biomedical sciences. Behav Res Methods 39(2):175–191. 10.3758/BF03193146 17695343 10.3758/bf03193146

[CR10] Fink A, Koschutnig K, Zussner T, Perchtold-Stefan CM, Rominger C, Benedek M, Papousek I (2021) A two-week running intervention reduces symptoms related to depression and increases hippocampal volume in young adults. Cortex 144:70–81. 10.1016/j.cortex.2021.08.010 34653905 10.1016/j.cortex.2021.08.010

[CR11] Firth J, Stubbs B, Vancampfort D, Schuch F, Lagopoulos J, Rosenbaum S, Ward PB (2018) Effect of aerobic exercise on hippocampal volume in humans: a systematic review and meta-analysis. NeuroImage 166:230–238. 10.1016/j.neuroimage.2017.11.007 29113943 10.1016/j.neuroimage.2017.11.007

[CR12] Frodl T, Strehl K, Carballedo A, Tozzi L, Doyle M, Amico F, Gormley J, Lavelle G, O’Keane V (2020) Aerobic exercise increases hippocampal subfield volumes in younger adults and prevents volume decline in the elderly. Brain Imaging Behav 14(5):1577–1587. 10.1007/s11682-019-00088-6 30927200 10.1007/s11682-019-00088-6

[CR13] Fuchs R, Klaperski S, Gerber M, Seelig H (2015) Measurement of physical activity and sport activity with the BSA questionnaire. Z fur Gesundheitspsychologie 23(2):60–76. 10.1026/0943-8149/a000137

[CR14] Gao Y, Syed M, Zhao X (2023) Mechanisms underlying the effect of voluntary running on adult hippocampal neurogenesis. Hippocampus 33(4):373–390. 10.1002/hipo.23520 36892196 10.1002/hipo.23520 PMC10566571

[CR23] Garber CE, Blissmer B, Deschenes MR, Franklin BA, Lamonte MJ, Lee IM, Swain DP (2011) Quantity and quality of exercise for developing and maintaining cardiorespiratory, musculoskeletal, and neuromotor fitness in apparently healthy adults: guidance for prescribing exercise. 10.1249/MSS.0b013e318213fefb 10.1249/MSS.0b013e318213fefb 21694556

[CR15] Gerber M, Brand S, Elliot C, Holsboer-Trachsler E, Pühse U, Beck J (2013) Aerobic exercise training and burnout: a pilot study with male participants suffering from burnout. BMC Res Notes 6(1):78. 10.1186/1756-0500-6-78 23497731 10.1186/1756-0500-6-78 PMC3599602

[CR16] Gianfredi V, Blandi L, Cacitti S, Minelli M, Signorelli C, Amerio A, Odone A (2020) Depression and objectively measured physical activity: a systematic review and meta-analysis. Int J Environ Res Public Health 17(10):3738. 10.3390/ijerph17103738 32466242 10.3390/ijerph17103738 PMC7277615

[CR17] Gray JP, Müller VI, Eickhoff SB, Fox PT (2020) Multimodal abnormalities of brain structure and function in major depressive disorder: a meta-analysis of neuroimaging studies. Am J Psychiatry 177(5):422–434. 10.1176/appi.ajp.2019.19050560 32098488 10.1176/appi.ajp.2019.19050560 PMC7294300

[CR18] Guadalupe T, Mathias SR, VanErp TGM, Whelan CD, Zwiers MP, Abe Y, Abramovic L, Agartz I, Andreassen OA, Arias-Vásquez A, Aribisala BS, Armstrong NJ, Arolt V, Artiges E, Ayesa-Arriola R, Baboyan VG, Banaschewski T, Barker G, Bastin ME, Francks C (2017) Human subcortical brain asymmetries in 15,847 people worldwide reveal effects of age and sex. Brain Imaging Behav 11(5):1497–1514. 10.1007/s11682-016-9629-z 27738994 10.1007/s11682-016-9629-z PMC5540813

[CR19] Hautzinger M, Bailer M, Hofmeister D, Keller F (2012) Allgemeine Depressionsskala (ADS). Manual 2. Auflage. Hogrefe Verlag GmbH, Göttingen

[CR20] Heissel A, Heinen D, Brokmeier LL, Skarabis N, Kangas M, Vancampfort D, Stubbs B, Firth J, Ward PB, Rosenbaum S, Hallgren M, Schuch F (2023) Exercise as medicine for depressive symptoms? A systematic review and meta-analysis with meta-regression. Br J Sports Med 57(16):1049–1057. 10.1136/bjsports-2022-106282 36731907 10.1136/bjsports-2022-106282 PMC10423472

[CR21] Hofmann P, Tschakert G (2017) Intensity-and duration-based options to regulate endurance training. Front Physiol 8:239374. 10.3389/fphys.2017.00337 10.3389/fphys.2017.00337 PMC5442222 28596738

[CR22] Hu S, Tucker L, Wu C, Yang L (2020) Beneficial effects of Exercise on Depression and anxiety during the Covid-19 pandemic: a narrative review. Front Psychiatry 11:587557. 10.3389/fpsyt.2020.587557 33329133 10.3389/fpsyt.2020.587557 PMC7671962

[CR24] IBM Corp. Released (2021) *IBMstatisticsistics for Windows*, Version 28.0. IBM Corp, Armonk, NY

[CR25] Iglesias JE, Augustinack JC, Nguyen K, Player CM, Player A, Wright M, Roy N, Frosch MP, McKee AC, Wald LL, Fischl B, van Leemput K (2015) A computational atlas of the hippocampal formation using ex vivo, ultra-high resolution MRI: application to adaptive segmentation of in vivo MRI. NeuroImage 115:117–137. 10.1016/j.neuroimage.2015.04.042 25936807 10.1016/j.neuroimage.2015.04.042 PMC4461537

[CR26] Iglesias JE, van Leemput K, Augustinack J, Insausti R, Fischl B, Reuter M (2016) Bayesian longitudinal segmentation of hippocampal substructures in brain MRI using subject-specific atlases. NeuroImage 141:542–555. 10.1016/j.neuroimage.2016.07.020 27426838 10.1016/j.neuroimage.2016.07.020 PMC5026967

[CR27] Kong XZ, Postema MC, Guadalupe T, de Kovel C, Boedhoe PSW, Hoogman M, Mathias SR, van Rooij D, Schijven D, Glahn DC, Medland SE, Jahanshad N, Thomopoulos SI, Turner JA, Buitelaar J, van Erp TGM, Franke B, Fisher SE, van den Heuvel OA, Francks C (2022) Mapping brain asymmetry in health and disease through the ENIGMA consortium. Hum Brain Mapp 43(1):167–181. 10.1002/hbm.25033 32420672 10.1002/hbm.25033 PMC8675409

[CR28] Lammer L, Beyer F, Luppa M, Sanders C, Baber R, Engel C, Wirkner K, Loffler M, Riedel-Heller SG, Villringer A, Witte AV (2023) Impact of social isolation on grey matter structure and cognitive functions: a population-based longitudinal neuroimaging study. eLife 12:1–65. 10.7554/eLife.83660 10.7554/eLife.83660 PMC10281670 37337666

[CR29] Laukkanen RMT, Kukkonen-Harjula TK, Oja P, Pasanen ME, Vuori IM (2000) Prediction of change in maximal aerobic power by the 2-km walk test after walking training in middle-aged adults. Int J Sports Med 21(2):113–116. 10.1055/s-2000-8872 10727071 10.1055/s-2000-8872

[CR30] Li H, Zhang X, Bi S, Cao Y, Zhang G (2022) Psychological benefits of green exercise in wild or urban greenspaces: a meta-analysis of controlled trials. Urban Forestry Urban Green 68:127458. 10.1016/j.ufug.2022.127458

[CR31] Liu HK (2022) Human adult hippocampal neurogenesis is back. Again? Cell Res 32(9):793–794. 10.1038/s41422-022-00698-8 35856092 10.1038/s41422-022-00698-8 PMC9436931

[CR32] Maller JJ, Broadhouse K, Rush AJ, Gordon E, Koslow S, Grieve SM (2018) Increased hippocampal tail volume predicts depression status and remission to anti-depressant medications in major depression. Mol Psychiatry 23(8):1737–1744. 10.1038/mp.2017.224 29133948 10.1038/mp.2017.224

[CR33] Martland R, Korman N, Firth J, Stubbs B (2023) The efficacy of exercise interventions for all types of inpatients across mental health settings: a systematic review and meta-analysis of 47 studies. J Sports Sci 41(3):232–271. 10.1080/02640414.2023.2207855 37132599 10.1080/02640414.2023.2207855

[CR34] Massazza A, Kienzler H, Al-Mitwalli S, Tamimi N, Giacaman R (2023) The association between uncertainty and mental health: a scoping review of the quantitative literature. J Mental Health 32(2):480–491. 10.1080/09638237.2021.2022620 10.1080/09638237.2021.2022620 35014927

[CR35] McEwen BS (2006) Protective and damaging effects of stress mediators: central role of the brain. Dialog Clin Neurosci 8(4):367–381. 10.31887/DCNS.2006.8.4/bmcewen 10.31887/DCNS.2006.8.4/bmcewen PMC3181832 17290796

[CR36] Nabkasorn C, Miyai N, Sootmongkol A, Junprasert S, Yamamoto H, Arita M, Miyashita K (2006) Effects of physical exercise on depression, neuroendocrine stress hormones and physiological fitness in adolescent females with depressive symptoms. Eur J Public Health 16(2):179–184. 10.1093/eurpub/cki159 16126743 10.1093/eurpub/cki159

[CR37] Nogovitsyn N, Muller M, Souza R, Hassel S, Arnott SR, Davis AD, MacQueen GM (2020) Hippocampal tail volume as a predictive biomarker of antidepressant treatment outcomes in patients with major depressive disorder: a CAN-BIND report. Neuropsychopharmacology 45(2):283–291. 10.1038/s41386-019-0542-1 31610545 10.1038/s41386-019-0542-1 PMC6901577

[CR38] Oja P, Mänttäri A, Pokki T, Kukkonen-Harjula K, Malmberg LR, Miilunpalo J, S., Suni J (2013) *UKK WALK TEST Tester’s guide* (4th revised edition). UKK Institute for Health Promotion Research

[CR39] Pearce M, Garcia L, Abbas A, Strain T, Schuch FB, Golubic R, Kelly P, Khan S, Utukuri M, Laird Y, Mok A, Smith A, Tainio M, Brage S, Woodcock J (2022) Association between Physical Activity and Risk of Depression: a systematic review and Meta-analysis. JAMA Psychiatry 79(6):550–559. 10.1001/jamapsychiatry.2022.0609 35416941 10.1001/jamapsychiatry.2022.0609 PMC9008579

[CR40] Pedisic Z, Shrestha N, Kovalchik S, Stamatakis E, Liangruenrom N, Grgic J, Titze S, Biddle SJH, Bauman AE, Oja P (2020) Is running associated with a lower risk of all-cause, cardiovascular and cancer mortality, and is the more the better? A systematic review and meta-analysis. Br J Sports Med 54(15):898–905. 10.1136/bjsports-2018-100493 31685526 10.1136/bjsports-2018-100493

[CR41] Radloff LS (1977) The CES-D scale: a self-report Depression Scale for Research in the General Population. Appl Psychol Meas 1(3):385–401. 10.1177/014662167700100306

[CR42] Reuter M, Schmansky NJ, Rosas HD, Fischl B (2012) Within-subject template estimation for unbiased longitudinal image analysis. NeuroImage 61(4):1402–1418. 10.1016/j.neuroimage.2012.02.084 22430496 10.1016/j.neuroimage.2012.02.084 PMC3389460

[CR43] Roddy DW, Farrell C, Doolin K, Roman E, Tozzi L, Frodl T, O’Hanlon E (2019) The hippocampus in depression: more than the sum of its parts? Advanced hippocampal substructure segmentation in depression. Biol Psychiatry 85(6):487–497. 10.1016/j.biopsych.2018.08.021 30528746 10.1016/j.biopsych.2018.08.021

[CR44] Roozendaal B, McGaugh JL (2011) Memory modulation. Behav Neurosci 125(6):797–824. 10.1037/a0026187 22122145 10.1037/a0026187 PMC3236701

[CR45] Saygin ZM, Kliemann D, Iglesias JE, van der Kouwe AJW, Boyd E, Reuter M, Stevens A, van Leemput K, McKee A, Frosch MP, Fischl B, Augustinack JC (2017) High-resolution magnetic resonance imaging reveals nuclei of the human amygdala: manual segmentation to automatic atlas. NeuroImage 155:370–382. 10.1016/j.neuroimage.2017.04.046 28479476 10.1016/j.neuroimage.2017.04.046 PMC5557007

[CR46] Sheline YI (2011) Depression and the hippocampus: cause or effect? Biol Psychiatry 70(4):308. 10.1016/j.biopsych.2011.06.006 21791257 10.1016/j.biopsych.2011.06.006 PMC3733566

[CR47] Sun Y, Hu N, Wang M, Lu L, Luo C, Tang B, Lui S (2023) Hippocampal subfield alterations in schizophrenia and major depressive disorder: a systematic review and network meta-analysis of anatomic MRI studies. J Psychiatry Neurosci 48(1):E34–E49. 10.1503/jpn.220086 36750240 10.1503/jpn.220086 PMC9911126

[CR48] Thomas AG, Dennis A, Rawlings NB, Stagg CJ, Matthews L, Morris M, Kolind SH, Foxley S, Jenkinson M, Nichols TE, Dawes H, Bandettini PA, Johansen-Berg H (2016) Multi-modal characterization of rapid anterior hippocampal volume increase associated with aerobic exercise. NeuroImage 131:162–170. 10.1016/j.neuroimage.2015.10.090 26654786 10.1016/j.neuroimage.2015.10.090 PMC4848119

[CR49] Travis SG, Coupland NJ, Hegadoren K, Silverstone PH, Huang Y, Carter R, Malykhin NV (2016) Effects of cortisol on hippocampal subfields volumes and memory performance in healthy control subjects and patients with major depressive disorder. J Affect Disord 201:34–41. 10.1016/j.jad.2016.04.049 27162154 10.1016/j.jad.2016.04.049

[CR50] Vilagut G, Forero CG, Barbaglia G, Alonso J (2016) Screening for depression in the general population with the center for epidemiologic studies depression (CES-D): a systematic review with meta-analysis. PLoS ONE 11(5) Article e0155431. 10.1371/journal.pone.0155431 10.1371/journal.pone.0155431 PMC4868329 27182821

[CR51] Voss MW, Nagamatsu LS, Liu-Ambrose T, Kramer AF (2011) Exercise, brain, and cognition across the life span. J Appl Physiol (Bethesda Md: 1985) 111(5):1505–1513. 10.1152/japplphysiol.00210.2011 10.1152/japplphysiol.00210.2011 PMC3220305 21527670

[CR52] Wilckens KA, Stillman CM, Waiwood AM, Kang C, Leckie RL, Peven JC, Foust JE, Fraundorf SH, Erickson KI (2021) Exercise interventions preserve hippocampal volume: a meta-analysis. Hippocampus 31(3):335–347. 10.1002/hipo.23292 33315276 10.1002/hipo.23292 PMC11497212

[CR53] World Health Organization [WHO] (2002) World health report: 2002—Reducing risks, promoting healthy life. Geneva, Switzerland. *World Health Organization*. https://www.who.int/publications/i/item/9241562072

[CR54] World Health Organization [WHO] (2020) *WHO guidelines on physical activity and sedentary behaviour: at a glance.* Geneva: World Health Organization; 2020. Licence: CC BY-NC-SA 3.0 IGO

[CR55] World Health Organization [WHO] (2022) *The World Mental Health Report: transforming mental health for all. *World Health Organization. https://www.who.int/publications/i/item/9789240049338

[CR56] Zajner C, Spreng RN, Bzdok D (2022) Lacking social support is associated with structural divergences in hippocampus-default network co-variation patterns. Soc Cognit Affect Neurosci 17(9):802–818. 10.1093/scan/nsac006 35086149 10.1093/scan/nsac006 PMC9433851

[CR57] Zakariás G, Petrekanits M, Laukkanen R (2003) Validity of a 2-km walk test in predicting the maximal oxygen uptake in moderately active Hungarian men. Eur J Sport Sci 3(1):1–8. 10.1080/17461390300073104

