# Supplementary material for: Changes in hippocampal volume and affective functioning after a moderate intensity running intervention
Source: Brain Struct Funct. 2024 Dec 13;230(1):2. doi: 10.1007/s00429-024-02885-2 (PMC11645311; doi:10.1007/s00429-024-02885-2)
Supplement: Supplementary file 1 — Supplementary Material 1 [file 429_2024_2885_MOESM1_ESM.docx]

**Supplementary Material**

**Title: Changes in hippocampal volume and affective functioning after a moderate intensity running intervention**

**Journal: Brain Structure and Function**

**Table 1**

***Volumes of the three main hippocampal regions and significant subfields (bilaterally)***

|  | left | | right | |
| --- | --- | --- | --- | --- |
| Subfields | *M* | *SE* | *M* | *SE* |
| **Hippocampal tail**  t_1_ | 620.29 | 13.51 | 629.53 | 18.43 |
| t_2_ | 613.41 | 13.74 | 629.77 | 17.63 |
| t_3_ | 620.55 | 14.26 | 638.17 | 19.21 |
| t_4_ | 619.36 | 13.62 | 627.73 | 16.73 |
| **Hippocampal body** |  |  |  |  |
| t_1_  t_2_ | 1228.59  1220.30 | 23.86  23.84 | 1225.23  1225.74 | 22.75  21.81 |
| t_3_ | 1228.11 | 23.93 | 1229.10 | 22.44 |
| t_4_  *GC ML DG body*  t_1_  t_2_  t_3_  t_4_ | 1220.55  133.28  131.42  134.75  131.95 | 23.10  3.17  3.19  3.18  2.99 | 1223.16  173.89  174.84  173.94  173.86 | 20.79  5.31  4.95  5.57  5.34 |
| **Hippocampal head** |  |  |  |  |
| t_1_  t_2_ | 1228.59  1220.30 | 23.86  23.84 | 1225.23  1225.74 | 22.75  21.81 |
| t_3_ | 1228.11 | 23.93 | 1229.10 | 22.44 |
| t_4_  *HATA*  t_1_  t_2_  t_3_  t_4_ | 1220.55  79.07  79.86  78.47  77.72 | 23.10  2.81  2.51  2.67  2.44 | 1223.16  77.43  78.22  77.62  76.42 | 20.79  2.22  2.30  1.92  1.85 |

*Note.* ***M* = Estimated Marginal Mean**, *SE* = Standard Error of mean. Hippocampal volume is measured in cubic millimeters (mm^3^). Volumetric changes for the three main hippocampal regions (head, body, tail) are included. Of the three main regions only the hippocampal tail showed a significant TIME effect. Significant TIME effects were also present for the left HATA and left GC-ML-DG-body.
